# Deubiquitinating enzymes (DUBs): Regulation, homeostasis, and oxidative stress response

**DOI:** 10.1016/j.jbc.2021.101077

**Published:** 2021-08-12

**Authors:** Nathan A. Snyder, Gustavo M. Silva

**Affiliations:** Department of Biology, Duke University, Durham, North Carolina, USA

**Keywords:** DUB, deubiquitination, ubiquitin, enzymatic regulation, oxidative stress, redox signaling, translation, A20, tumor necrosis factor alpha–induced protein 3, AMSH, associated molecule with the SH3 domain of STAM, ATXN, ataxin, BAP1, BRCA1-associated protein 1, BER, base excision repair, CK2, casein kinase 2, CYLD, conserved cylindromatosis, DTT, dithiothreitol, DUB, deubiquitinating enzyme or deubiquitinase, EGFR, epidermal growth factor receptor, ESCRT, endosomal sorting complexes required for transport, FOXO4, forkhead box O4, ISR, integrated stress response, MDM2, Mouse double minute 2, MIC-CAP, microcephaly-capillary malformation, MINDY, motif interacting with Ub-containing novel DUB, MJD, Machado–Josephin domain, NLS, nuclear localization signal, OTU, ovarian tumor domain, OTUB, varian tumor deubiquitinase, ubiquitin aldehyde binding, OTUD, ovarian tumor deubiquitinase, PCNA, proliferating cell nuclear antigen, Polβ, DNA polymerase beta, PTM, posttranslational modification, QC, quality control, ROS, reactive oxygen species, RPN11, regulatory particle non-ATPase 11, RQC, ribosome-associated quality control, RTU, redox control of translation by ubiquitin, SUMO, small ubiquitin-like modifier, TGF-β, transforming growth factor beta, TNF, tumor necrosis factor, TRAF2, tumor necrosis factor (TNF) receptor associated factor 2, TRAF6, tumor necrosis factor (TNF) receptor associated factor 6, Ubp, ubiquitin-binding protein, UCH, ubiquitin C-terminal hydrolase, UIM, ubiquitin interacting motif, UPS, ubiquitin-proteasome system, USP, ubiquitin-specific protease, WDR48, WD repeat domain 48

## Abstract

Ubiquitin signaling is a conserved, widespread, and dynamic process in which protein substrates are rapidly modified by ubiquitin to impact protein activity, localization, or stability. To regulate this process, deubiquitinating enzymes (DUBs) counter the signal induced by ubiquitin conjugases and ligases by removing ubiquitin from these substrates. Many DUBs selectively regulate physiological pathways employing conserved mechanisms of ubiquitin bond cleavage. DUB activity is highly regulated in dynamic environments through protein–protein interaction, posttranslational modification, and relocalization. The largest family of DUBs, cysteine proteases, are also sensitive to regulation by oxidative stress, as reactive oxygen species (ROS) directly modify the catalytic cysteine required for their enzymatic activity. Current research has implicated DUB activity in human diseases, including various cancers and neurodegenerative disorders. Due to their selectivity and functional roles, DUBs have become important targets for therapeutic development to treat these conditions. This review will discuss the main classes of DUBs and their regulatory mechanisms with a particular focus on DUB redox regulation and its physiological impact during oxidative stress.

When the subject of ubiquitination arises, most people think of the canonical pathway of ubiquitin-dependent proteasomal targeting. This, however, only represents a fraction of the functional diversity of ubiquitin. Ubiquitin is a small (76 amino acid) and highly conserved eukaryotic protein that acts as a posttranslational protein modifier (1). Ubiquitin signaling is a very robust and diverse process where a series of ubiquitin conjugases and ligases act in a coordinated process to covalently modify targets with one or more ubiquitin molecules in the form of a chain (2, 3, 4). Each of these different ubiquitin chains has a unique structure, thus providing the opportunity for this single protein to influence numerous pathways, other than proteasomal degradation, from DNA damage repair to protein translation and trafficking (2, 5, 6, 7, 8, 9). In most of these pathways, including protein degradation, ubiquitin is removed from its substrates in a reversible fashion in a process achieved through dynamic regulation of ubiquitin hydrolases known as deubiquitinating enzymes (DUBs) (10, 11, 12). Due to the highly adaptive and reversible nature of ubiquitin signaling, DUBs are frequently utilized to regulate protein function in response to environmental changes and stress (13, 14, 15, 16).

In the canonical ubiquitination pathway (Fig. 1), an E1 ubiquitin activating enzyme is responsible for activating ubiquitin in an ATP-dependent manner and charging the E2 ubiquitin conjugase with the ubiquitin (17). The E2 then either transfers the ubiquitin to the substrate or to an E3 ubiquitin ligase that will then transfer the ubiquitin molecule to the substrate (18). In the former case, the substrate is brought into proximity of the E2 through an associated E3 ubiquitin ligase (18). A number of E2 and E3 ubiquitin enzymes exist (∼40 E2s and over 600 E3s in humans and 12 E2s and ∼80 E3s in yeast), and the pairing of these enzymes determines the specificity of substrate that is ubiquitinated (19, 20). This pairing is also responsible for determining how the ubiquitin chains are assembled (18, 21).

**Figure 1:**
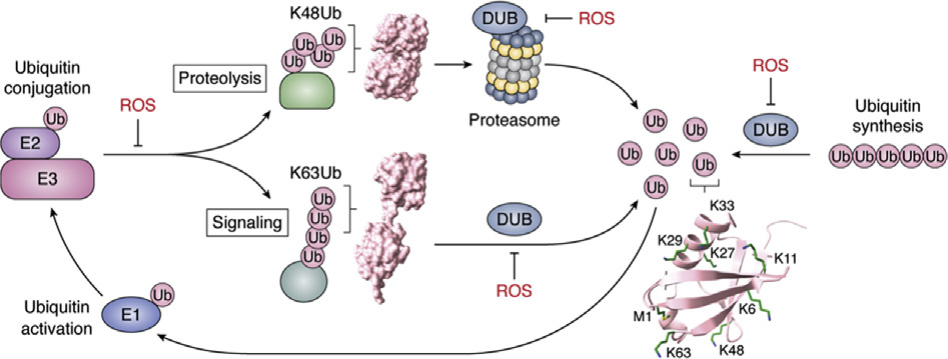
**Cycle of ubiquitin signaling.** Ubiquitin (ub) is synthesized as polymers or fusions of ribosomal proteins that are cleaved into ubiquitin monomers by deubiquitinating enzymes (DUBs). Ubiquitin monomers are then used by E1 ubiquitin-activating enzymes to charge E2 ubiquitin conjugases, which work with or without E3 ubiquitin ligases to attach ubiquitin to targets. The conformation of K-linked ubiquitin chains (*e.g.*, K48 or K63) determines the fate of the targets, whether they undergo ubiquitin signaling, or if they are sent to the proteasome for degradation. In either case, DUBs are responsible for removal of the ubiquitin and replenishment of the ubiquitin monomer pool. Reactive oxygen species (ROS)-sensitive steps are labeled in red. Structures depicted include ubiquitin (PDB: 1UBQ), and representatives of two forms of di-ubiquitin, K48 (PDB: 3AUL) and K63 (PDB: 3H7P).

Protein ubiquitination most commonly occurs *via* an isopeptide bond between the C-terminus of ubiquitin and a lysine of the substrate (22). Other forms of nonlysine ubiquitination are rare but exist, including peptide bonds with the N-terminal methionine (M1), thioester bonds with a cysteine residue, and hydroxyester bonds with a serine or threonine residue (23). Furthermore, ubiquitin has recently been shown to be conjugated to nonprotein surfaces such as lipopolysaccharides (24). Once the first ubiquitin molecule is conjugated to a substrate, a second round of conjugation occurs linking two ubiquitin molecules together in the form of a chain (25). Ubiquitin is linked into polyubiquitin chains through any of the seven lysine residues in ubiquitin (K6, K11, K27, K29, K33, K48, or K63) as well as through the amino group of M1 (26). Each of these linkage sites provides a unique chain archetype, and while many of these chains can signal for proteasome-dependent substrate degradation to some extent, others serve an array of regulatory roles (26).

Proteomics data demonstrate that while most ubiquitin linkage types increase upon inhibition of the proteasome, K63-linked polyubiquitin does not, suggesting that it is involved almost exclusively in nonproteasomal pathways (27, 28). Each linkage forms unique topologies that may be bound and recognized by distinct proteins to generate different signals and functions (29). Additionally, ubiquitin can form both homotypic chains, where each ubiquitin is linked through the same lysine residue (*e.g.*, K48-linked chains or K63-linked chains) and heterotypic chains, where multiple linkage types are utilized to form the chain (30). Furthermore, ubiquitin can be assembled to produce branched chains and can themselves be modified through PTMs such as phosphorylation or SUMOylation, further expanding the signaling possibilities (30, 31).

While the regulation of ubiquitin conjugation has been more widely studied, significant advancement in the study of the family of enzymes responsive for removal of ubiquitin has only recently occurred. Humans encode ∼100 different DUBs that regulate ubiquitin signaling by removing ubiquitin and thus disassembling the chains and thereby their signals, while recycling ubiquitin for further conjugation (32). This can be done by cleaving single ubiquitin monomers from the distal end of a chain or by cleaving entire chains by breaking the bond between the proximal ubiquitin and the substrate (12).

Similar to ubiquitin ligases and conjugases, DUBs usually have specific ubiquitin linkage types that they bind as substrates (11). Since DUBs serve as direct antagonists of ubiquitin conjugation, this results in a switch-like system (11). The levels of ubiquitin conjugation can therefore be determined by these two competing enzymatic systems, which can themselves be regulated at expression, subcellular location, or activity levels through mechanisms controlled by protein–protein interactions and posttranslational modifications (PTMs) (Fig. 2) (33, 34).

**Figure 2:**
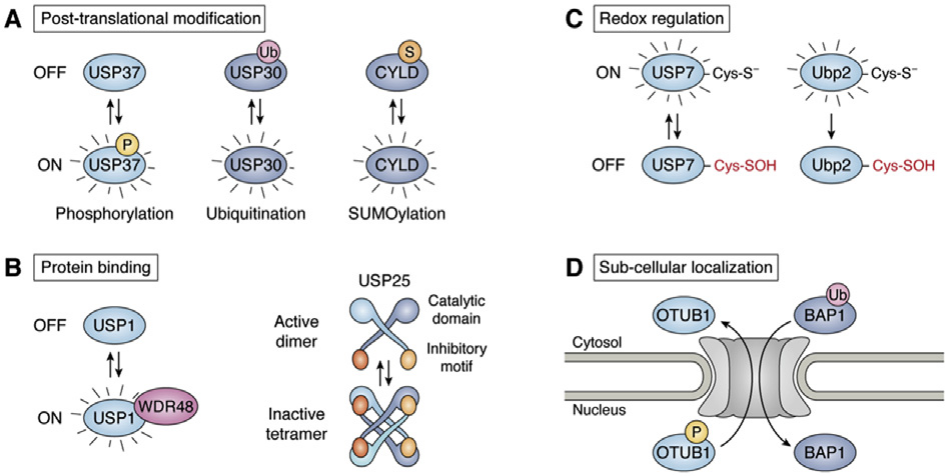
**DUB regulatory mechanisms.** Examples of posttranslational DUB regulatory mechanisms are depicted. These regulatory mechanisms include: (*A*) posttranslational modification (*e.g.*, ubiquitination, SUMOylation, phosphorylation), (*B*) protein binding, (*C*) redox regulation, and (*D*) subcellular localization.

E2s, E3s, and DUBs can respond to environmental cues that induce the prioritization of the generation or removal of signals (3). DUBs in particular are highly sensitive to environmental stresses, and this review will discuss the main mechanisms of DUB regulation, in particular those that result from oxidative stress, which directly targets and inhibits the active site of a large number of DUBs (35).

The ability of DUBs to regulate such a dynamic signaling system also makes them an interesting target for therapeutics. Indeed, dysregulation or malfunction of DUBs has been implicated in a number of human diseases, including cancers and neurological disorders, usually resulting from aberrant signaling within the cell (36, 37). Due to their physiological importance, a number of small-molecule inhibitors of selective DUBs are being developed with the goal of therapeutic utilization as treatments for these diseases (38). Recent insights into the mechanisms and importance of DUB regulation, combined with the development of drugs with therapeutic potential, have marked the importance of this newly burgeoning field and provided directions for future studies to come.

## WHY do we need DUBs?

As we learn more about the importance and diversity of ubiquitin chains in cellular processes, we also gain emphasis on the importance of the DUBs that regulate these signals. In addition to regulating the ubiquitin signals generated by E2s and E3s, DUBs are essential to maintain the supply of ubiquitin by recycling and breaking down newly synthesized ubiquitin fusion proteins (Fig. 1) (39, 40). Constant maintenance of a free ubiquitin pool is necessary for cellular homeostasis but also required, to rapidly generate signals to respond to changes in the cellular environment or to biomolecular damage incurred during stress (41). This section will highlight the classes of DUBs and the mechanisms and functional roles they play in eukaryotic cells. Table 1 summarizes the class, family, and functional roles of all DUBs specifically discussed in this review.

**Table 1:** List of DUBs mentioned in this review and their classifications

DUB (^∗^known to be redox-sensitive)	Class	Family	Known ub linkage specificity	Known pathways affected
A20^∗^	Cysteine protease	out	K63	NF-κB signaling (157)
AMSH	Metalloprotease	JAMM/MPN+	K63	Endocytosis/sorting (249)
AMSH-LP	Metalloprotease	JAMM/MPN+	K63	Endocytosis/sorting (249)
ATXN3	Cysteine protease	MJD	K48, K63	Protein homeostasis (250, 251); ER-associated degradation (252); transcription regulation (253); cytoskeletal regulation (254); DNA repair (255)
ATXN3L	Cysteine protease	MJD	K48, K63	Protein homeostasis (256)
BAP1	Cysteine protease	UCH	K48	DNA repair/transcription (257, 258)
BRCC36	Metalloprotease	JAMM/MPN+	K63	DNA repair/cell cycle (259, 260)
Cezanne^∗^	Cysteine protease	out	K11, K48, K63	NF-κB signaling (261)
CSN5	Metalloprotease	JAMM/MPN+	K63	DNA repair (262); cell cycle (263); protein sorting (264)
CYLD	Cysteine protease	out	K63, M1	Cell cycle (265); NF-κB/WNT signaling (266, 267)
JOSD1	Cysteine protease	MJD	K48, K63	Endocytosis; membrane sorting (268)
JOSD2	Cysteine protease	MJD	K48, K63	Metabolism (269)
MINDY1	Cysteine protease	MINDY	K48	Self-renewal of stem cells (270)
MINDY2	Cysteine protease	MINDY	Non-specific	
MINDY3	Cysteine protease	MINDY	K48	
MINDY4	Cysteine protease	MINDY	K48	
MINDY4B	Cysteine protease	MINDY	K48	
MYSM1	Metalloprotease	JAMM/MPN+	K63	Transcription (271); immune signaling (272)
OTUB1^∗^	Cysteine protease	out	K48	DNA repair (273); immune signaling (274)
OTUB2	Cysteine protease	out	K11, K48, K63	DNA repair (275); protein homeostasis (276); translation (124)
OTUD1^∗^	Cysteine protease	out	K63	Translation (124); immune signaling (277)
OTUD2^∗^	Cysteine protease	out	K11, K27, K29, K33, K48, K63	Protein sorting; ER unfolded protein response (278)
OTUD3^∗^	Cysteine protease	out	K6, K11, K27, K48	Translation (124); immune regulation (279)
OTUD5^∗^	Cysteine protease	out	K48, K63	Cell signaling (280, 281); immune regulation (282)
Otulin	Cysteine protease	out	M1	NF-κB/WNT signaling (283)
RPN11	Metalloprotease	JAMM/MPN+		Proteasome (81)
Ubp2^∗^	Cysteine protease	USP	K63	DNA repair (284); translation (121); protein sorting (146)
Ubp3	Cysteine protease	USP		Protein sorting (285); translation (129)
UCHL1^∗^	Cysteine protease	UCH		MAP kinase pathway (286); translation (124)
UCHL3^∗^	Cysteine protease	UCH	K48	Insulin signaling (287); protein sorting (288)
UCHL5^∗^	Cysteine protease	UCH	K48	DNA repair (289); proteasome (83)
USP1^∗^	Cysteine protease	USP	monoubiquitin	DNA repair (290)
USP4^∗^	Cysteine protease	USP	K48, K63	RNA splicing (291); immune response (292); signaling (167, 168, 293)
USP5^∗^	Cysteine protease	USP	K48	DNA repair (294); immune response (295)
USP7^∗^	Cysteine protease	USP	K48, K63	Autophagy (296); protein transport (114); DNA repair (297); signaling (298)
USP8^∗^	Cysteine protease	USP	K48, K63	Protein sorting (299); cell cycle (300); DNA repair (301)
USP10^∗^	Cysteine protease	USP		Autophagy (172); DNA repair (302); signaling (303); translation (128)
USP12	Cysteine protease	USP		Autophagy (304); cell cycle (305)
USP14^∗^	Cysteine protease	USP		DNA repair (306); proteasome (54); chemotaxis (307)
USP19^∗^	Cysteine protease	USP	K63	Cell cycle (308); DNA repair (309); ER-associated degradation (310)
USP21^∗^	Cysteine protease	USP		Cell cycle (311); transcription (312); translation (124)
USP25^∗^	Cysteine protease	USP	K48, K63	ER-associated degradation (313); signaling (314, 315); trafficking (316)
USP28^∗^	Cysteine protease	USP		DNA repair (317); cell cycle (318)
USP32	Cysteine protease	USP		Cell proliferation (319); trafficking (113)
USP37^∗^	Cysteine protease	USP	K11, K48	Cell cycle (320); DNA replication (321)
USP46	Cysteine protease	USP		Signaling (322, 323)
USP47	Cysteine protease	USP		DNA repair (120); cell cycle (324)
USP9X	Cysteine protease	USP	K29, K33, K48	Cell cycle (325); signaling (326)
ZUFSP	Cysteine protease	ZUFSP	K11, K48, K63	DNA repair and replication (66)

Abbreviations: BRCC36, BRCA1/BRCA2-containing complex subunit 3; CSN5, COP9 signalosome complex subunit 5; JOSD, Josephin domain-containing; MYSM1, Myb-like, SWIRM and MPN domains 1; ZUFSP, zinc finger with UFM1-specific peptidase domain.

### DUB classification

There are two major classes of DUBs, cysteine proteases and metalloproteases (42, 43). The former of these classes is further broken down into six families of proteins, based on sequence conservation and domain architecture (44). However, all DUBs in this class utilize a catalytic triad composed of an active site cysteine residue, along with a histidine and (in most cases) an asparagine or aspartate, to catalyze the hydrolysis of the ubiquitin linkages (43). Because of this catalytic cysteine, this class of DUBs can be regulated by reactive oxygen species (ROS) during oxidative stress, adding to the complexity of mechanisms by which DUBs may be regulated (15, 35, 45). Instead of a catalytic cysteine, metalloprotease DUBs catalyze isopeptide hydrolysis *via* a catalytic serine and a zinc ion cofactor (42, 46). While the activity of DUBs from both families can be regulated, this difference in catalytic mechanism makes metalloproteases more resistant to oxidative stress (15). The characteristics of both the cysteine protease and metalloprotease classes of DUBs are discussed further below.

#### Cysteine proteases

The cysteine protease class of DUBs is by far the most well studied of the two. This is due not only to their greater number, with over 80 known in humans, but also to them being the first DUBs to be structurally and mechanistically characterized, which led to the development of inhibitors designed to target the catalytic site (47, 48, 49). Having inhibitors to this class of DUBs enabled faster identification and characterization of new members, thus resulting in better knowledge of their impact on cellular physiology and how they might be used in the manipulation of therapeutic systems (49). The six families of cysteine protease DUBs include the ubiquitin C-terminal hydrolase (UCH), ubiquitin-specific protease (USP), ovarian tumor (OTU), Machado–Josephin domain (MJD), K48 polyubiquitin-specific MINDY domain families, as well as the newest-discovered DUB family, zinc finger with UFM1-specific peptidase domain, named for its founding protein member (44).

The UCH family of DUBs possesses four mammalian members (UCHL1, UCHL3, UCHL5, and BAP1) with UCHL3 being the first DUB to be structurally characterized (47). A primary feature of DUBs from the UCH family is a loop structure that covers the active site, limiting the size of substrate with which they can interact to small peptides, such as those that result from proteasomal or lysosomal degradation (47, 50). Some larger substrates, however, may be accommodated through unfolding (50). One UCH DUB, BAP1, is involved in regulation of the cell cycle and DNA damage response (51). Germline mutations in BAP1, causing loss of activity, result in a predisposition to malignant tumors, such as malignant melanoma or renal cell carcinoma (52).

**Figure 3:**
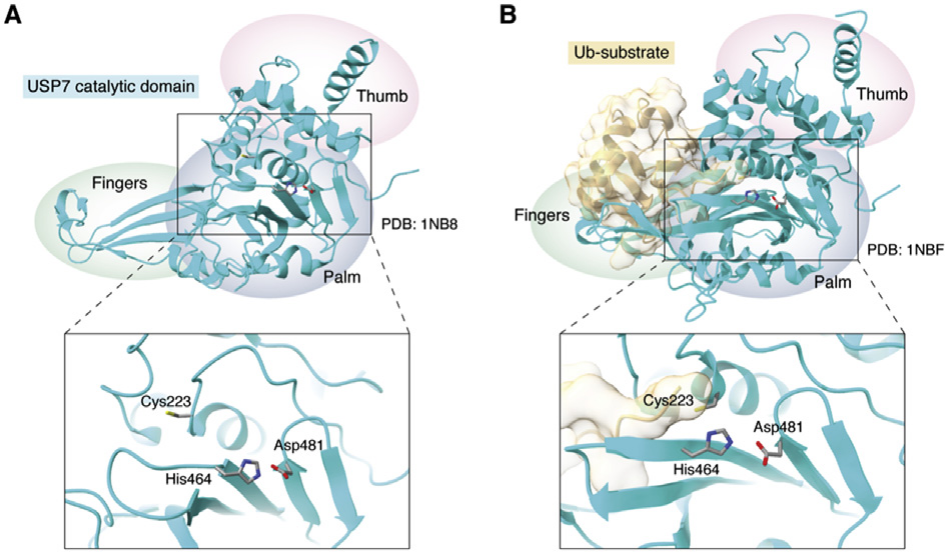
**Conformations of cysteine proteases.** Inactive (*A*, PDB: 1NB8) and active (*B*, PDB: 1NBF) conformations of the USP family cysteine protease USP7 are shown in *cyan*. Below are enlarged views of the catalytic triad positions. In the inactive state (*A*), the catalytic cysteine (Cys223) is positioned far from the other members of the catalytic triad (His464 and Asp481). This prevents the histidine from lowering the pKa to deprotonate the thiol of the cysteine and promote the active state. In the active state (*B*), USP7 is bound to a ubiquitin substrate, which induces a conformational shift that brings the catalytic cysteine closer to the other members of the catalytic triad, enabling its deprotonation into a reactive thiolate. In either case, the fingers domain (containing the ubiquitin interaction motif), as well as the palm and thumb domains (containing the catalytic center) are indicated.

The USP family of DUBs is the largest, comprised of 58 members in the human genome. Members of this family have a structure described as being in the form of a hand, with three subdomains: the thumb, palm, and fingers (Fig. 3) (53, 54, 55). The catalytic site sits between the thumb and palm subdomains, while the fingers are responsible for stabilizing the interaction with distal ubiquitin on substrates (53).

There are 17 members of the OTU family of DUBs, most of which display linkage specificity for ubiquitin substrates (56). For example, ovarian tumor deubiquitinase, ubiquitin aldehyde binding (OTUB1) is specific for K48 chains, while Cezanne is specific for K11 chains, and ovarian tumor deubiquitinase (OTUD2) demonstrates activity for K11, K27, and K33 chains (56, 57, 58). This specificity is achieved through use of additional ubiquitin interaction sites that enable binding to longer chains of specific linkages, and it has led to OTU DUBs being known for regulating signaling pathways (56). Another interesting aspect of the OTU family of DUBs is that some lack the asparagine or aspartate residue of the catalytic triad in DUBs (59). While some of these, like OTUB2, are predicted to be inactive, resulting from the absence of the negatively charged member of the catalytic triad to polarize the histidine, tumor necrosis factor alpha–induced protein 3 (A20) has been demonstrated to retain activity after an induced mutation of its catalytic aspartate residue (60, 61).

The four members of the MJD family (ataxin 3 [ATXN3], ATXN3L, Josephin domain-containing 1, and Josephin domain-containing 2) all present a highly conserved catalytic Josephin domain, which contains two ubiquitin binding sites as well as two conserved histidines, along with the catalytic cysteine, that are necessary for catalysis (44, 62). This family is named for Machado–Josephin disease, a neurological disorder caused by an expansion of the CAG repeat motif in ATXN3, producing polyglutamine and causing protein misfolding and aggregation (63). They are completely absent in yeast, presumably evolving to function in higher eukaryotes.

The MINDY family of DUBs, which contains five members (MINDY1–4 and MINDY4B), has a unique catalytic triad of Cys, His, and Gln and is highly specific for K48 ubiquitin linkages (44, 64). The members of this family have also been demonstrated to be autoinhibited prior to substrate binding, which causes a conformational shift to activate the enzyme (65).

Finally, the newest family, zinc finger with UFM1-specific peptidase domain, contains only one member of the same name, which has two ubiquitin-binding domains, both of which are required for its highly specific cleavage of K63 linkages (66). Despite the variations in domain architectures and amino acid sequences, all of these families of DUBs have a catalytic core comprised of a cysteine and a histidine residue (44, 67).

**Figure 4:**
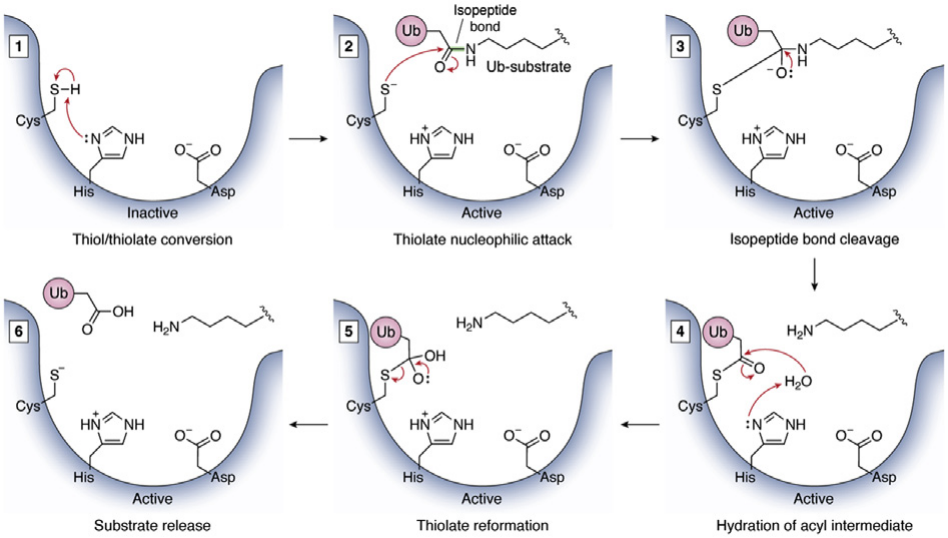
**DUB cysteine protease catalytic mechanism.** A generalized mechanism of cysteine protease DUBs is shown, including the three most common members of the catalytic triad (Cys, His, and Asp) and the isopeptide bond of the ubiquitinated substrate. The general steps of the mechanism are as follows: 1, the histidine, depolarized by the aspartate, deprotonates the cysteine, converting its side chain from an inactive thiol to a reactive thiolate. 2, the thiolate of cysteine undergoes a nucleophilic attack on the acyl group of the ubiquitin isopeptide bond, forming a tetrameric intermediate. 3, the isopeptide bond is cleaved as the amide group of the isopeptide bond deprotonates the DUB histidine, freeing the substrate from ubiquitin, which is still bound as an intermediate with the DUB cysteine. 4, hydration of the DUB cysteine acyl intermediate utilizing a water molecule to convert the acyl intermediate to a carboxyl intermediate. 5, the intermediate bond between the ubiquitin and DUB cysteine is broken, reforming the ubiquitin monomer and thiolate. 6, the ubiquitin monomer and substrate are released from the DUB.

Mechanistically, cysteine protease enzymes rely on a reactive cysteine residue in the catalytic site (Fig. 3) (44, 67). This active/inactive state of cysteine protease DUBs is dependent on whether the cysteine contains an inactive thiol (-SH) or reactive thiolate (-S^−^) group (68). Transition between these states usually occurs through conformational shifts caused by substrate binding, affiliation with a protein complex, or PTM of the DUB itself (Fig. 3*B*) (32, 53, 69, 70). This conformational shift results in the polarization of the active site histidine, often achieved by the presence of an asparagine or aspartate residue (59, 71). This polarization lowers the pKa of the cysteine, causing deprotonation of the thiol and stabilization of the thiolate (Fig. 4) (10, 67). Once in the active thiolate state, the cysteine is able to undergo a nucleophilic attack on the isopeptide bond linking ubiquitin to its substrate or on the polyubiquitin chain, forming a thioester intermediate with the substrate, prior to release and reactivation of the DUB (Fig. 4) (10, 72). Substrate release occurs when the DUB hydrolyzes the bonds of the ubiquitin/DUB intermediate, and the DUB resets back to a thiolate, ready to begin a new enzymatic cycle (10, 72).

One reason proposed for the inactive conformation of cysteine proteases is to protect the DUBs from oxidation by maintaining the cysteine in the inactive thiol form (35). This does not leave the cysteine immune to oxidation, but it does lessen its reactivity, and therefore may offer some amount of protection from oxidative stress. Because of the reactivity of cysteines with ROS, all cysteine proteases, including DUBs, are potentially susceptible to inhibition, due to covalent modification in the active site (73). This means that, during times of oxidative stress, a large number of DUBs may be rapidly inhibited through cysteine oxidation, increasing the amount of ubiquitin conjugation that occurs throughout the cell. During homeostasis, a number of substrates are regularly ubiquitinated and deubiquitinated (74). In response to oxidative stress, accumulation of ubiquitin conjugates will derive from inhibition of DUBs, based on their varied ROS sensitivity, leading to modulation of pathways in accordance with cellular needs for oxidative stress response (35). In addition, other targets might accumulate due to proteasome inhibition (75) or increased activity of E2s and E3s (76). Although E2s and E3s are themselves enzymes containing a catalytic cysteine, only two yeast E2s, out of 12 known to exist, have been shown to be regulated under oxidative stress, adding to the balance of ubiquitin conjugates (77, 78). However, the regulation of these enzymes is also only beginning to be elucidated.

#### Metalloproteases

The metalloprotease class of DUBs are comprised of zinc-dependent enzymes with JAB1/MPN/MOV34 (JAMM/MPN+) domains (42, 79). The human genome encodes 14 genes with this domain, of which seven are capable of coordinating the catalytically required zinc ion and only six have been demonstrated to possess the ability to hydrolyze ubiquitin conjugates (AMSH, AMSH-LP, BRCA1/BRCA2-containing complex subunit 3, COP9 signalosome complex subunit 5, Myb-like, SWIRM and MPN domains 1, and regulatory particle non-ATPase 11 [RPN11]) (46, 59, 80).

One of the earliest and best-studied examples of DUB metalloproteases is RPN11, one of three DUBs associated with the proteasome (the other two are the cysteine proteases USP14 and UCHL5) (81, 82, 83). Used to destroy and recycle damaged or unwanted proteins from the cell, the proteasome, a 26S protein complex, is comprised of a barrel-shaped 20S catalytic core, which is capped on one or both ends by different regulatory particles (84, 85). While the 20S core particle is responsible for carrying out proteolysis of substrates, the 19S regulatory particle provides the high specificity for ubiquitinated substrates, through ubiquitin-binding receptors (85, 86). Prior to substrate degradation, the polyubiquitin chain is removed and recycled to the pool of free ubiquitin (87). RPN11 cleaves the link between the substrate and the proximal ubiquitin of the chain, thus removing the entire polyubiquitin chain at once (81, 87).

The activity of RPN11 is dependent on its interaction with the proteasome as well as the proteasome ATPase activity responsible for unfolding of the substrate (88, 89). This dependency on substrate unfolding delays the activity of RPN11 until the substrate is fully committed to degradation in the proteasome (88). Failure of RPN11 to remove the polyubiquitin chain results in steric hindrance of the substrate into the 20S core particle and thus prevents protein degradation, making RPN11 essential to the ubiquitin-proteasome system (UPS) (88). Meanwhile, the cysteine proteases USP14 and UCHL5, the other DUBs of the proteasome, instead serve a regulatory role, trimming the ubiquitin from substrates to limit their degradation (82, 90).

Another metalloprotease DUB, AMSH, is involved in regulating endosomal membrane trafficking by removing K63-linked ubiquitin from cargoes (91, 92). The activity of AMSH determines whether substrate cargoes are ultimately degraded or recycled by the ESCRT pathway (92). This is a critical pathway for neuronal development, and mutations in AMSH have been linked to the developmental disorder microcephaly-capillary malformation (MIC-CAP) syndrome, making AMSH an important target for therapeutic treatment (93).

The catalytic sites of the DUB metalloprotease class contain an aspartate, a serine, and two histidine residues (42, 46). The catalytic site also requires a zinc ion, which is coordinated by the aspartate and histidine residues, as well as a water molecule. The mechanism of action by the DUBs in this class utilizes a zinc ion to generate a hydroxide ion from water to hydrolyze the isopeptide bond between ubiquitin and substrate (42). This mechanism means that the DUBs in this class never form a covalent intermediate between the enzyme and substrate, making them resistant to classical DUB inhibitors as well as redox reactions, which typically covalently target the catalytic cysteine of the other DUB families to inhibit DUB activity (11).

### Cellular functions regulated by DUBs

We have thus far presented a general overview of DUBs and the catalytic mechanisms through which they remove ubiquitin signals from substrates. However, DUBs do not only function to counteract ubiquitin signals. One of the most important functions of DUBs is to recycle and produce free ubiquitin (41). This maintains a pool of ubiquitin from which the various ubiquitin enzymes may draw to target proteins for degradation or signaling (41). The largest portion of this pool comes from both *de novo* synthesis of ubiquitin and from recycling of ubiquitin at the proteasome from proteins targeted for degradation (41).

In terms of protein synthesis, ubiquitin is unique in that it is not synthesized as a single protein (94). Instead, it is encoded either with a C-terminally fused ribosomal protein, as is the case with UBA52 and UBA80 in mammals (Ubi1–3 in yeast), or as a ubiquitin polymer linked in a head-to-tail fashion followed by a variable length C-terminus (95, 96, 97). UBA52 is a fusion of ubiquitin and the ribosomal protein eL40 while UBA80 is a fusion of ubiquitin and eS31 (96, 98). For this reason, several DUBs, including UCHL3, USP9X, USP7, USP5, and Otulin, are required to hydrolyze these proteins releasing free ubiquitin monomers (94). For the C-terminal fusion proteins, this occurs posttranslationally, while for ubiquitin polymers, it may occur either posttranslationally or cotranslationally (94).

Besides the production of free ubiquitin monomers from protein fusions and polymers, DUBs also recompose the pool of free ubiquitin by recycling it from substrates destined to degradation by the proteasome, as mentioned above (41). Proteasome-associated DUBs, RPN11, USP14, and UCHL5, cleave the ubiquitin from the substrate, which is then unfolded by proteasomal ATPases and passed into the 20S core particle for degradation (85). USP14 (ubiquitin-binding protein [Ubp6] in yeast) and UCHL5 are cysteine proteases that bind to the regulatory particle of the proteasome and function to trim ubiquitin from substrates, potentially rescuing proteins from degradation (99). Since RPN11 resides within the core particle in humans and yeast, it removes ubiquitin from substrates already committed to degradation (81). These DUBs thereby replenish the pool of free ubiquitin, rather than allow them to be degraded along with the substrate (41). Since many DUBs are sensitive to oxidative stress, this can impact the DUB-dependent replenishment of the ubiquitin pool by simultaneously inhibiting DUBs that function at the synthesis and recycling steps (Fig. 1) (41). Without DUB-dependent replenishment of ubiquitin monomers, the amount of free ubiquitin would rapidly decrease as ubiquitin conjugation reactions occur, leading to limitation on the rapidity of ubiquitin signaling and widespread regulation of cellular pathways necessary to survival (74).

Another function of DUBs is to regulate the ubiquitin signaling network and thereby control a number of important cellular processes, including protein trafficking, DNA damage repair, and translation regulation (74, 100, 101, 102). These processes often require rapid and dynamic changes due to shifts in the cellular environment. Through modulation of DUB activity, proteins can be destabilized or stabilized rapidly through addition and removal of ubiquitin, respectively (10). This is particularly useful during cellular response to stress, where regulatory mechanisms occur, leading to activation or inhibition of DUBs required to stabilize stress factors or foster the degradation of unneeded proteins (11). Some highlights of the role of DUBs in these processes will be briefly discussed below.

#### Protein trafficking

Ubiquitin signaling plays a quite prominent role in trafficking (100, 103). Studies have shown that at the plasma membrane, ubiquitination occurs on adaptor proteins or even directly on cargoes to impact internalization (8, 104, 105). Destabilization of adaptors by ubiquitin blocks internalization by preventing assembly of the core components of the endocytic machinery around the cargo (8). In contrast, ubiquitination of the cargoes induces internalization, targeting them to the endosome and, eventually, to destruction at the lysosome (106, 107, 108, 109). In this case, DUBs function to clear ubiquitin from the cargo, preventing its degradation and allowing it to undergo recycling to the surface (103, 110). This is particularly important in cell surface receptor activation, as DUBs maintain the duration of signaling by preventing the degradation of cell surface receptor, as is the case with the DUB USP8 and epidermal growth factor receptor (EGFR) (111, 112). DUBs, such as USP32 and USP7, can also influence intracellular membrane protein trafficking by similar methods (113, 114).

#### DNA damage repair

DUBs function to regulate the DNA damage repair system, making it readily available during times of damage and stress (101, 115). For example, under normal conditions, phosphorylated USP7 stabilizes the E3 ubiquitin ligase MDM2 (Mouse double minute 2), which ubiquitinates and targets p53 for degradation (116, 117). Upon stress, USP7 is inactivated by dephosphorylation, which destabilizes MDM2, leading to increased levels of p53 (118). DUBs, like Ubp8 of the SAGA (Spt-Ada-Gcn5 acetyltransferase) complex, also regulate the ubiquitination of histones and other DNA-binding proteins to increase or decrease access of stress/damage response elements to DNA (119). Finally, DUBs also play a role in stabilizing some of the response elements themselves, as is the case with USP47 and DNA polymerase β (Pol β), which is responsible for the DNA single-strand break and base excision repair mechanisms (120).

#### Translation

DUBs have also been found to influence translation by regulating ubiquitination of ribosomes themselves (102). This places DUBs in an important position to regulate global protein synthesis. Recently, a new form of DUB regulation of ribosomes, termed RTU (redox control of translation by ubiquitin), has been discovered in yeast (9, 121). In the RTU, a DUB central to the pathway, Ubp2, becomes reversibly inhibited by ROS during oxidative stress (121). This inhibition results in accumulation of K63-linked polyubiquitin chains on ribosomes mediated by the E2 Rad6 and E3 Bre1, leading to a buildup of polysomes, suggesting that this modification arrests translation elongation (77, 121, 122, 123). Interestingly, deletion of *UBP2* from yeast was found to increase the K63 ubiquitin signaling, even in the absence of oxidative stress (121). This suggests that the K63 ubiquitination of the ribosome by Rad6/Bre1 may occur constitutively, but it is tightly regulated by Ubp2 (121).

K63-ubiquitinated ribosomes from cells undergoing oxidative stress are predominately isolated in a rotated pretranslocation state, suggesting that this stage of translation is lengthened or arrested (122). As such, it is likely that ubiquitination of ribosomes occurs to reduce global translation during oxidative stress to reduce the synthesis of unneeded proteins and prevent accumulation of damaged ones. Other instances of DUBs influencing translation have been identified as well. USP21 and OTUD3 antagonize the ubiquitination of eS10 and uS10 that results from integrated stress response (ISR) and ribosome-associated quality control pathway (RQC) (124). These responses are induced by ribosome stalling, which occurs due to amino acid deprivation or defective mRNAs that lack 3′UTRs or stop codons (125). Ribosome ubiquitination by ISR and RQC results in ribosome dissociation and degradation of the associated mRNA and arrested peptide (125, 126, 127).

The small subunit of the ribosome, which is ubiquitinated during quality control processes, is rescued from degradation by the activity of the DUB USP10, which removes ubiquitin from the ribosomal proteins eS10, uS3, and uS5, allowing recycling of the small subunit for new translation reactions (128). Other DUBs are also involved in this quality control pathway, including OTUB2, OTUD1, and UCHL1 although their roles are not yet fully understood (124). Finally, Ubp3 and Otu2 in yeast are required for efficient translation (129). Ubp3 is responsible for inhibiting polyubiquitination of eS7 by the E3 Hel2, which would trigger RQC and suppress translation (129). The activity of Ubp3 leaves eS7 in the monoubiquitinated form (129). Otu2 deubiquitinates eS7 in order to promote dissociation of the small ribosomal subunit from the mRNA upon completion of translation (129). This cycling of ubiquitination/deubiquitination demonstrates the intricacy and importance of DUB regulation of fundamental cellular pathways.

Not only do DUBs regulate translation elongation, but they also regulate translation initiation and ribophagy. USP9X and USP11 have been demonstrated to stabilize the initiation factor eIF4A and eIF4B, respectively (130, 131). These initiation factors bind the 5′-UTR of mRNA as part of a complex and unwind its secondary structure to enable the binding of the small ribosomal subunit (132). Beyond their direct role in protein synthesis, DUBs are also involved in ribophagy. Ribophagy is a process, directed by ubiquitination, in which ribosomal subunits are targeted for degradation, particularly as a result of nutrient starvation (133). This process was identified in yeast and found to be regulated through the activity of the DUB Ubp3 (the yeast homolog of USP10), in coordination with Bre5 (133). The existence of multiple DUB-regulated pathways that are targeted directly to the translation machinery emphasizes the need for further research in these areas, since translation regulation is a critical component of cellular activity and stress response.

## Regulation of DUB activity

We have discussed so far a number of cellular processes that are regulated by DUBs in eukaryotes. These processes rely on the specificity of individual DUBs regarding their substrate and linkage type but also on the intrinsic regulation of their activity. Because DUBs are a large family of proteins, there is variability in the mechanisms by which they are regulated. Generally, DUBs function to antagonize the ubiquitination of substrates carried out by E2 and E3 enzymes (32). Therefore, the relative abundance and activity of both DUBs and ubiquitin enzymes act as a switch-like mechanism (11). For this reason, small shifts in either the population of ubiquitin enzymes or DUBs can cause large differences in whether the ubiquitinated or deubiquitinated population of a substrate is predominant (11). The regulation of DUBs is an emerging field of study with much remaining to be discovered as scientists uncover the structures and pathways influenced by individual enzymes. Like most proteins, DUBs can be regulated by altering gene expression and protein localization. Here, however, we will review two other categories of mechanisms by which the activity of DUBs is directly regulated: allosteric regulation by protein binding and PTMs, including the redox regulation of the cysteine protease DUBs (Fig. 2).

### Protein binding

Protein–protein interactions are an important aspect of DUB regulation. Some DUBs function as components of large complexes, and they require the binding of the other complex members to function (12). In the most well-known example, the three DUBs, RPN11, USP14, and UCHL5, bind to the proteasome to deubiquitinate proteins substrates destined to degradation, thus recycling the ubiquitin molecules (99). For each of these DUBs, interaction with the proteasome complex is required for them to achieve an active state, as it causes conformational shifts that enable substrate binding and activation of the catalytic site (81, 134, 135).

The requirement for proteasomal DUBs to interact with the proteasome complex to gain activity is a critical regulatory element for cells, because without it, the DUBs would be able to remove the ubiquitin signals from protein targets destined for proteasomal degradation prior to target arrival at the catalytic subunits (90). Premature deubiquitination would lead to substrate stabilization due to escape of degradation processes. In conditions where a number of misfolded and damaged proteins need to be removed to prevent cytotoxic aggregation, such as induced stress, dysregulation of DUBs could be highly detrimental to cellular viability (136). Therefore, binding of individual adapter proteins to DUBs can control access to the substrate, complex interactions, and conformational shifts between active and inactive states (137). In this dynamic system, this regulation plays a key role in specificity of time and location of DUB activity to maintain tight control of ubiquitin signaling.

Some DUBs also function through interactions with smaller complexes. WD Repeat Domain 48 (WDR48) is a required cofactor that activates the DUBs USP1, USP12, and USP46 (138, 139). Structural analysis has demonstrated that the WD repeat β propeller of WDR48 interacts with the distal end of the fingers domain of USP46 (140). Mutational analysis suggests the same is true of WDR48 interaction and activation of USP1 (140). Interestingly, this binding enhances the activity of these DUBs by increasing the rate of substrate turnover without enhancing substrate affinity (140). Another binding partner for USP12, WDR20, also has a WD repeat β propeller, but it binds to a different site than WDR48 on USP12 to cause allosteric activation (141, 142).

One particularly interesting mechanism of DUB activity regulation through protein binding is that of USP25, which forms a homotetramer to inhibit cleavage of ubiquitinated substrates (143). In the active state, USP25 forms a homodimer where two subunits interact through an inhibitory loop domain (143). This inhibitory loop has affinity for the ubiquitin-binding domain of other USP25 homodimers and thus can bind and form a tetramer, where each homodimer is bound and inhibited by the inhibitory loop of the other (143) (Fig. 2*B*). Formation of this tetramer is inhibited by a phosphomimetic mutation of Tyr454 to glutamate, suggesting a posttranslational mechanism to drive USP25 toward the dimer conformation, but this mechanism has not yet been confirmed (143). Another DUB, USP28, forms similar active homodimers, but lacks the formation of the inhibited homotetramer (144, 145).

Some protein interactions with DUBs do not alter activity, but rather provide specificity. In yeast, the DUB Ubp2 possesses catalytic activity, but requires an adapter protein, Rup1, to bind to and regulate the activity of the E3 ubiquitin ligase, Rsp5 (146, 147, 148). This Ubp2-Rup1 interaction is required for deubiquitination and destabilization of adapter proteins that promote interaction between Rsp5 and substrates (146, 147, 148). Protein binding of DUBs not only brings them into larger protein complexes, but it can also induce specificity and alter their active state, either through allosteric modification of the catalytic or ubiquitin-binding domains, or through competitive inhibition of those sites (33, 149). This enables use of small-molecule inhibitors to modulate DUB activity for the purposes of research or therapeutics for diseases caused by deregulation of DUBs.

Due to the large array of DUBs present in most species and the variety of signaling pathways and conditions that may impact DUB interactions, there remains a significant amount of research to be done to understand the molecular details governing the interaction network of DUB-binding partners and the physiological impact of those interactions. Further understanding of these interactions could facilitate a greater insight into not only the function and substrates of individual DUBs but also the impacts and regulation of unique ubiquitin signaling pathways.

### Posttranslational modification

Another mechanism frequently employed to regulate DUB activity is through PTMs (Fig. 2). These covalent modifications are usually reversible and can cause an increase or decrease in the activity of DUBs, impact their subcellular localization, or alter chain specificity of the DUBs (150). Without these regulatory mechanisms in place, the homeostasis of ubiquitin signaling would be thrown out of balance, thus impacting a number of cellular processes (12). As other types of DUB regulation, some of these modifications trigger DUBs to stabilize proteins while others inhibit DUB activity, thus increasing levels of protein ubiquitination and degradation (Fig. 2) (150). In response to environmental stresses or metabolic needs, DUB regulation by PTMs can induce the activation of the appropriate response or rapidly shut down cellular processes at the protein level (2, 32). Described below are some of the common PTMs of DUBs and the effects of the modifications that have been identified.

#### Phosphorylation

DUB phosphorylation can have major impacts on DUB function, but also on the cell itself. For example, USP37 regulates the cell cycle by deubiquitinating and stabilizing cyclin A, which enables cells to enter into S phase (151, 152). USP37 is phosphorylated by CDK2 during the G1/S-phase transition (151). The exact mechanism for the activation of USP37 by phosphorylation is not yet fully understood, but since the phosphorylated residue, Ser628, is in close proximity to the ubiquitin interacting motif (UIM) domain repeats of USP37, it is presumable that phosphorylation causes a structural rearrangement that enhances the affinity for substrates (151, 153). Confirming the importance of Ser628 phosphorylation for USP37 activity, phosphomimetic mutation of this residue (Ser628Asp) confers a higher amount of DUB activity than a nonphosphorylatable mutant (Ser628Ala) (151). Other DUBs are known to be activated by phosphorylation, including USP1, USP14, OTUD5, and A20, which play roles in DNA damage repair, proteasome-based degradation, interferon production, and NF-κB signaling, respectively, to name a few (154, 155, 156, 157, 158, 159, 160).

To contrast this, some DUBs are also inhibited by phosphorylation. This is the case for conserved cylindromatosis (CYLD), a tumor suppressor responsible for stabilization of tumor necrosis factor (TNF) receptor associated factors 2 and 6 (TRAF2 and TRAF6) (161, 162). Upon phosphorylation at Ser418 by NF-κB kinase subunit ε (IKKε), CYLD loses its catalytic efficiency, which leads to increased degradation of TRAF2 and TRAF6 (163, 164). A point mutation at this site confirms the effect of phosphorylation on CYLD activity (164). USP8 is one of multiple DUBs that regulate trafficking by removing ubiquitin from endocytic cargoes, stimulating cargo recycling from the endosome rather than further trafficking to the lysosome for degradation (111). Its activity is similarly inhibited by phosphorylation of Ser680, enhancing the ubiquitination and degradation of internalized EGFR to prevent further receptor activation (165).

Besides the regulation of DUB catalytic activity, phosphorylation can also mediate the subcellular relocalization of DUBs. For example, phosphorylation of OTUB1 at Ser16 by casein kinase 2 (CK2) causes it to translocate from the cytosol to the nucleus (166). In contrast, phosphorylation of USP4 at Ser445 by protein kinase B (AKT) causes translocation from the nucleus to the cytosol (167). In the nucleus, USP4 functions to regulate Wnt signaling stabilizing β-catenin, enabling activation of downstream gene transcription (168). In the cytosol, USP4 impacts Wnt signaling by blocking activation of Disheveled (Dvl), but it also exerts influence on the NF-κB, TGF-β, and p53 signaling pathways (167, 169, 170, 171). USP4 is not alone among DUBs that undergo relocalization as a result of phosphorylation events.

Phosphorylation of USP10 and ATXN3, which regulate p53 stability, also results in nuclear localization (172, 173). Each of these DUBs undergoes nuclear localization resulting from phosphorylation caused by CK2 or ataxia telangiectasia mutated (ATM) kinase activity upregulated in response to DNA damage, such as that caused by ionizing radiation or oxidative stress (174, 175). Nuclear translocation regulated by phosphorylation is usually achieved by a conformational shift that exposes a nuclear localization signal (NLS) (176). However, the exact mechanism of the nuclear translocation of these DUBs has not yet been characterized. ATXN3 does have a NLS, although it remains uncertain whether this is required for nuclear uptake (177). The downstream signaling pathways influenced by the localization of these DUBs emphasize the potential of DUB PTMs to have widespread cellular implications.

#### Ubiquitination/SUMOylation

Although ubiquitination of proteins is traditionally linked with their degradation by the proteasome, which regulates activity by breaking down the enzyme, others are regulated by nondegradative ubiquitin and ubiquitin-like signals, which can impact function and localization (178, 179). In the case of ubiquitination, many DUBs are often able to self-deubiquitinate to remove the regulation (Fig. 2) (180, 181, 182). Some of these modifications affect localization, moving the DUBs between subcellular compartments, and thus altering the availability of substrates. For example, BRCA1-associated protein 1 (BAP1) can be ubiquitinated near its nuclear localization sequence, allowing it to accumulate in the cytosol until it deubiquitinates itself and is translocated back to the nucleus, where it regulates cell proliferation by stabilizing transcription factors and chromatin-binding proteins that upregulate transcription (182).

Sometimes ubiquitin modifications affect activity by modulating the interaction between the DUB and its substrate. This interaction causes conformational shifts that affect the ubiquitin interaction domain or active site accessibility. Such is the case with USP30, which is monoubiquitinated at three sites near its substrate interaction domain by the E3 Parkin (183, 184, 185). Monoubiquitination of USP30 diminishes its catalytic activity to oppose Parkin-dependent activation of mitophagy, thus destabilizing mitochondria (185). USP30 is able to undergo autodeubiquitination to remove the modification (185). The regulation of USP30 activity is important, since hyperactivation of USP30 prevents Parkin from promoting removal of dysfunctional mitochondria (186, 187). Accumulation of these damaged organelles is a hallmark of Parkinson’s disease (188). Similarly, SUMO, a prominent ubiquitin-like protein, can be conjugated to the DUB CYLD at Lys40, a modification that does not impair substrate binding, but does reduce the ability of CYLD to deubiquitinate TRAF2 and TRAF6, promoting NF-κB signaling, and potentially leading to cell cycle dysregulation and cancer (189). For these reasons, both USP30 and CYLD are currently targets for therapeutic development to treat Parkinson’s disease and cancer (38, 190). Furthermore, the regulation of NF-κB signaling by CYLD has also been demonstrated to be important in preventing inflammation leading to diabetic nephropathy, adding to the therapeutic potential of CYLD (14).

Proteome-wide analyses have identified numerous PTM sites on DUBs that are phosphorylated, ubiquitinated, acetylated, or SUMOylated, but their functional role has not yet been characterized (191, 192, 193, 194). Further study of these modifications may help identify the function and regulation of these DUBs and the pathways they regulate.

#### Cysteine oxidation

The other PTMs described above are regularly discussed regarding their impact on enzymatic function, but the redox-dependent regulation of DUBs is an emerging and extremely relevant field that has gained increased attention in the last decade. Since many DUBs are cysteine proteases, they are often susceptible to regulation *via* oxidation of the catalytic cysteine (15, 195). Oxidation of catalytic cysteine was once considered in the category of protein damage; however, many enzymes are known to be reversibly regulated by redox reactions, which are critical to control cellular survival and adaptation oxidative stress (196).

**Figure 5:**
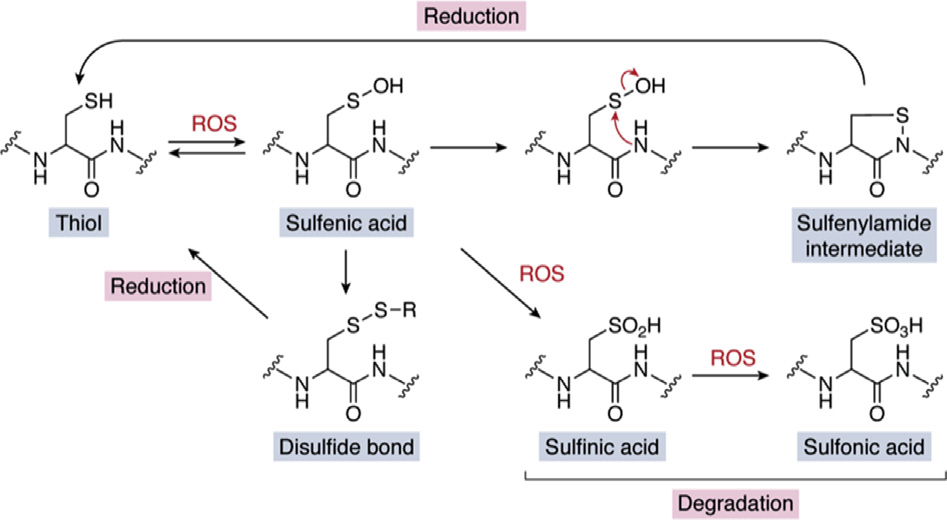
**States of DUB cysteines during reversible and irreversible oxidation resulting from redox regulation.** When subjected to reactive oxygen species (ROS), the catalytic cysteine of DUBs may be oxidized, converting the thiol/thiolate into different species. Primary oxidation reaction generates a reversible sulfenic acid. This reversibility can be stabilized by further conversion of the sulfenic acid into disulfide through hydrolysis with another cysteine thiol or through the generation of a sulfenylamide intermediate through a hydrolysis reaction with the amine group of the neighboring amino acid. Either of these states may be reduced to a thiol. In contrast, further oxidation of the sulfenic acid group results in the formation of irreversible sulfinic acid or sulfonic acid side chains, permanently inhibiting the DUB, requiring degradation of the oxidized DUB and *de novo* synthesis to reacquire activity.

Cysteine oxidation occurs in the presence of ROS, which react with the thiol/thiolate of cysteine to form the reversible sulfenic acid (-SOH), which could undergo conversion to a disulfide bond, through hydrolysis with another cysteine, or to a sulfenylamide intermediate, through hydrolysis with the amine group of a neighboring amino acid, (Fig. 5) (197, 198, 199, 200). All of these states are able to be reversed back to an active thiol through enzymatic reactions catalyzed by glutaredoxins or thioredoxins (Fig. 5) (198, 200). However, after further oxidation, the reversible sulfenic acid may be converted to the irreversible sulfinic acid (-SO_2_H) or sulfonic acid (-SO_3_H) (Fig. 5) (200). These states cannot be reduced and require degradation for clearance and *de novo* synthesis of the DUB to restore activity (Fig. 5) (200).

Because the cysteine proteases require a deprotonated and reactive thiolate (-S^−^) in the catalytic cysteine to function, oxidation of their thiol group renders the DUBs inactive (195). This leaves them particularly vulnerable to oxidative stress caused by accumulation of ROS (195).

ROS are generated endogenously as part of incomplete reduction of oxygen molecules in the mitochondrial electron transport chain, but they may also be encountered through environmental factors such as exposure to pollution or radiation as well as lifestyle and diet (201, 202). Accumulation of ROS is usually counterbalanced by antioxidant enzymes, such as superoxide dismutases (SODs), peroxidases, and catalase, as well as small molecules such as glutathione, ascorbate, and tocopherol (203, 204, 205).

Oxidative stress occurs when ROS accumulate beyond the capacity of the cellular redox homeostasis systems (206). In response to stress, there is an accumulation of ubiquitinated proteins conjugated with distinct linkages (207). While oxidized and unneeded proteins are K48-ubiquitinated and degraded by the proteasome, K63-ubiquitin chains serve signaling functions and are later removed by DUBs, after the stress has been resolved (121, 207, 208). As cells age, ROS accumulation also becomes increasingly prevalent, and it has been linked to numerous human diseases, including neurodegenerative disorders, heart disease, complications with diabetes, and cancer (14, 209, 210, 211). These disorders are typically resultant from damage to DNA, RNA, and proteins that occurs as a result of ROS-mediated reactions (210).

Cysteine oxidation is unique to the other modifications that regulate DUB activity, because a single molecule, such as H_2_O_2_, can trigger the modification to impact a vast number of the DUBs within a cell, usually without the need for any enzymatic intermediates to perform the modification itself (35, 195). In particular, the members of the USP, OTU, and UCH families have been demonstrated to be reversibly inhibited by oxidative stress (15, 35, 45). Due to the rapid inhibition of DUBs that occurs from the direct targeting of the catalytic cysteines during oxidative stress, there is a drastic impact on ubiquitin signals, causing massive ubiquitin accumulation on proteins (35, 121, 212). This makes DUBs a primary cellular redox sensor, which leads to degradation of misfolded or damaged proteins and shutting down unnecessary metabolic and signaling pathways while relieving inhibition on those that are required for stress response (195).

When the thiol group of cysteine is oxidized by a single oxygen, it becomes sulfenic acid (-SOH), a modification that is easily reversed to regenerate the thiol (213). However, depending on the amount of ROS or the duration of stress, the redox inhibition of DUBs can have prolonged cellular effects. While the susceptibility of many DUBs to oxidative stress remains to be fully characterized, it has been established that many DUBs are reversibly inhibited by oxidative stress (15, 35, 45). The reversibility of the inhibitory oxidation suggests that these DUBs either stabilize the sulfenic acid or form inter- or intramolecular disulfide bonds. Formation of these structures might protect the cysteine from further oxidation until the stress is resolved and the cysteine can be reduced to reproduce the functional thiol.

DUBs were discovered more recently than the ubiquitinating enzyme, and their regulation is still not fully understood. In particular, regulation through oxidation has only been recently identified. The first example of reversible oxidation of DUBs was identified in Cotto-Rios *et al.* (2012), (15) where the authors demonstrated that cysteine protease family of DUBs and not the metalloprotease family were susceptible to oxidants, as expected, due to catalytic cysteine oxidation that was reversible by addition of the reducing agent dithiothreitol (DTT). The authors specifically identified USP1 and USP7 as being reversibly inhibited by this modification, and they observed accumulation of ubiquitin on their respective substrates, PCNA (Proliferating Cell Nuclear Antigen) and p53 (15). Mass spectrometry analysis determined that indeed it was the catalytic cysteine of these enzymes that was undergoing oxidation to inhibit enzymatic activity (15).

Shortly after the findings of Cotto-Rios *et al.* were published (15), two other independent groups confirmed and expanded upon the reversibly oxidized DUBs to include members of the OTU family as well as the USP and UCH families of DUBs (35, 45). Kulathu *et al.* (2013) (45) conducted crystallographic analysis of the oxidation of the A20 catalytic domain and found that the reversibly oxidized A20 had formed a cysteine sulfenic acid intermediate that was stabilized through hydrogen bonding to a neighboring Cys-loop, preventing further oxidation. They also demonstrated that some OTU DUBs are more resistant to oxidative stress than others, suggesting that DUB inhibition by oxidation does not occur universally as a switch, but rather more gradually as a dimmer, affecting certain DUBs and pathways at far lower concentrations than others (45).

Lee *et al.* (2013) (35) reported the interesting insight that some DUBs are resistant to oxidation while their catalytic site remains in the inactive state, with the cysteine in the thiol state, aimed away from the active site. They further showed that some DUBs that have been exposed to ROS can have different responses to reduction through DTT treatment, with some being activated by DTT (*e.g.*, USP8, USP10, and USP19), some having only enhancement of activity in the presence of DTT (*e.g.*, USP7, CYLD, and UCHL5), and others exhibiting no activity, despite the presence of a reducing agent (*e.g.*, USP1, USP22, and A20) (35). Although, in the latter case, this could be due to the need of a cofactor or coenzyme for full activity, as they found was the case with USP1, which required its activator UAF1 to regain activity (35). They observed that USP7, which has a disordered active site until bound to a substrate or cofactors, was resistant to oxidation unless exposed to a high pH or coincubated with ubiquitin (35). While high pH causes deprotonation of the cysteine thiol, coincubation with ubiquitin allosterically activates USP7 (Fig. 3), both processes causing an increase in sensitivity of USP7 to oxidation by ROS (35). Additionally, they found through mass spectrometry analysis that oxidation of the more oxidant-sensitive USP19CD led to the formation of a sulfenyl-amide intermediate between the catalytic cysteine (Cys506) and a neighboring serine (Ser510) (35). The formation of this intermediate is critical to the reversibility of the cysteine oxidation, as a Ser510Gly mutation abolished the restoration of activity following treatment with DTT (35).

Silva *et al.* (2015) (121) evaluated the role of oxidative stress in yeast and identified the DUB Ubp2 as a key regulator of K63 ubiquitination in the RTU pathway described above. *In vitro* analysis of Ubp2 confirmed that it is inhibited by the presence of hydrogen peroxide in a manner that was reversible by addition of the reducing agent, DTT (121). This suggests that Ubp2, like many other USP family proteins, is reversibly inhibited through oxidation, likely of its catalytic cysteine Cys745 (121, 148). During oxidative stress recovery in yeast, Ubp2, previously inhibited through oxidation, rapidly regains function, removing K63-linked ubiquitin from ribosomes and relieving its effect on translation (121). The rapid efficiency by which ubiquitin accumulates and is removed from ribosomes during oxidative stress suggests that the ubiquitin enzymes involved are constitutively active but regulated by Ubp2 (121). This means that, in this pathway, Ubp2 functions as a redox sensor to allow reduction of translation that is required for proper stress response (121).

One more example of redox regulation of DUB activity was presented in Kahles *et al.* (2021). The group showed that accumulation of K48 and K63 ubiquitin after ischemia reperfusion was caused primarily by redox inhibition of DUBs instead of inhibition of the proteasome (214). Although the DUBs involved in this pathway were not identified, the determination that redox regulation of intracellular DUBs and not proteasomal inhibition was responsible for polyubiquitin accumulation generates numerous opportunities for new lines of research into postischemic ubiquitination and human health (214).

## Cellular consequences of redox regulation of DUBs

In this review, we have discussed an important regulatory mechanism by which an array of DUBs could be regulated by ROS. Redox regulation of DUBs by ROS can serve as signaling mechanisms when present in a localized fashion or in low abundance but can also promote widespread control of cellular functions during oxidative stress (215). Considering the wide ranges of functions performed by ubiquitin signaling, global inhibition of DUBs during oxidative stress can simultaneously impact several distinct pathways in cellular physiology. In the context of oxidative stress, cells must regulate gene expression at transcription and translation as well as regulate metabolic, cell division, repair pathways, protein degradation, and many additional cellular processes to cope with this environmental aggression (206, 216). Many of these cellular functions are modulated by reversibly regulating DUB activity. Below are some examples of research that have already been reported to that end; however, this is an ever-expanding field that likely has numerous cellular impacts left to uncover.

One pathway that is known to be controlled by ubiquitin signaling is the trafficking of biomolecules within the cell. In response to stress, cells must regulate protein trafficking to stimulate stress response signaling and activity by relocalizing regulatory factors for action. Usp8 is a redox-sensitive DUB, and its depletion results in deregulated internalization of membrane receptors and the formation of large endosomes with ubiquitinated cargoes (217). Presumably, inhibition of Usp8 during oxidative stress induces internalization of cell surface receptors to prevent stimulation of signaling pathways until the stress is resolved. It is likely that the metalloprotease DUB AMSH, being a redox-resistant metalloprotease DUB, continues to function in order to promote some level of recycling of receptors, rather than degradation (15). This would result in cells maintaining lower levels of receptors at the surface, enabling them to be more prepared for the recovery of normal cellular activity as the stress is resolved. Understanding the cellular consequences of redox sensitivity variance in DUBs that perform similar functions could help us understand the evolutionary pressures that resulted in these differences.

Another hallmark of oxidative stress is the formation of stress granules (218, 219). Stress granules are large aggregate structures of RNA and protein that form as a result of several cellular stresses to regulate translation and to sequester and protect cytosolic proteins from the potentially damaging effects of stress (220). While ubiquitination is dispensable for their formation, ubiquitin can be found at the surface of stress granules, and regulation of ubiquitin signaling is required for efficient stress granule clearance during stress resolution (221). Redox-dependent inhibition of DUBs causes an increase in size and stability of stress granules, but during recovery from stress, DUBs are reactivated and remove the ubiquitin signaling from the stress granules, causing them to fragment and dissolve (221). Addition of DUB inhibitors during resolution of oxidative stress caused by sodium arsenite has been shown to increase the retention of stress granules significantly, with 75% of cells having stress granules present after 2 h of recovery, as opposed to 10% of cells in control samples (221). This emphasizes the importance of DUB activity during the recovery from oxidative stress, particularly as redox regulation of the DUBs themselves can therefore determine the duration of stress granule retention, depending on the speed of DUB oxidation reversal. Furthermore, both stress granule formation and oxidative stress have been implicated in neurological disorders such as amyotrophic lateral sclerosis and spinocerebellar ataxia type 2, and DUBs could therefore be a common element between both of these factors (25, 222, 223, 224, 225).

The effects of oxidative stress on DUB-dependent trafficking can have further downstream consequences, including influencing cell cycle regulation. This is the case with the DUB CYLD, which regulates translocation of NF-κB to the nucleus by inhibiting the IKK translocating complex through removal of K63-linked polyubiquitin (162, 226). By regulating the activation of NF-κB, CYLD therefore inhibits proinflammatory signaling, meaning disruption of CYLD through oxidative stress could enhance inflammation (227). Hyperactivation of NF-κB has also been implicated in the development and metastasis of cancer and even the resistance of cancer to radiation therapy (228, 229). While NF-κB has been a target of cancer therapeutics, perhaps CYLD, as an upstream regulator, could also be target for therapeutic development for cancers and inflammatory diseases (227).

Redox regulation of DUBs can also affect the cellular capacity to repair DNA damage. Upon DNA damage, such as thymine dimerization or apurinic/apyrimidinic (AP) sites, PCNA is monoubiquitinated by the E2 RAD6 and E3 RAD18 (230). This monoubiquitination is required for PCNA to recruit TLS (translesion synthesis) polymerases to bypass the stress-induced DNA lesion, and it is normally removed by the DUB USP1 (154). However, oxidative stress also inhibits USP1, allowing the TLS response to proceed (15). This inhibition of USP1 is rapid and reversible, allowing the cell to have a high tolerance for oxidative stress that would otherwise damage the DNA in replicating cells (15).

Another redox-sensitive DUB, USP7, also regulates the ubiquitination of PCNA, creating a redundant system to ensure resistance to oxidative stress (231). Interestingly, artificially elevated suppression of USP7 increases the mutation frequency resultant from oxidative stress, while the suppression of USP1 does not (231). This suggests that USP7 is more resistant to oxidative stress than USP1, and that it likely serves to regulate the TLS response to maintain fidelity of repair, emphasizing the importance of differential sensitivity of DUBs to oxidation (231). Supporting this is the modulation of the activity of the transcription factor FOXO4 by USP7 (232). FOXO4 is monoubiquitinated as a result of oxidative stress, causing its relocalization to the nucleus, where it undertakes transcriptional activity (232). USP7 modulates this activity by deubiquitinating FOXO4, causing nuclear export (232). In this way, FOXO4-stimulated DNA damage response is likely moderated unless ROS levels are high enough to inhibit USP7, triggering hyperactivation of FOXO4 (231, 232).

Lastly, a critical component of base excision repair (BER) is DNA polymerase β (polβ), which is constitutively ubiquitinated and targeted for degradation in the cytosol (120). Removal of the ubiquitin by USP47 is required to stabilize polβ and allow it to relocalize to the nucleus, where it carries out BER activity (120). The expression and activity of polβ are regulated in part by USP47 (120). USP47 overexpression, as well as underexpression, can lead to an increase of mutations and deficiency of DNA repair (120). While the ROS sensitivity of USP47 has not been directly assessed, it is likely that, as a cysteine protease, it bears some level of sensitivity, making it similar to USP7 in terms of modulation of oxidative stress response. Presumably, the dependence of DNA damage response on oxidation-sensitive DUBs suggests that the repair mechanisms could result in further damage if ROS levels are too high, leading to the inhibition of these stress-resistant DUBs. Further analysis is required to determine what levels of ROS would block DNA damage repair and whether the DUB activity and repair mechanisms resume as ROS levels are decreased to more moderate levels by cellular reductive systems.

During oxidative stress, cells are able to globally inhibit translation through activation of integrated stress response (ISR) while upregulating important genes involved in stress response and regulation of damaged protein and mRNA (233). The ribosome has been identified as a hub for ubiquitin, impacting its biogenesis, activity, and its role in quality control (QC) (9). Since ubiquitin signaling exerts such control over the ribosome, redox regulation can therefore have a major role in control of translation, by impacting every stage of the ribosome life cycle (9). Redox inhibition of the DUBs involved in quality control pathways could be detrimental. For example, inactivation of USP21, OTUD3, or USP10 all causes an increase in readthrough of stalling sequences, which could potentially contribute to an accumulation of aberrant proteins (124). In yeast, redox inhibition of Ubp2 during RTU enhances K63 ubiquitination of ribosomes, which further accumulates rapidly upon introduction of hydrogen peroxide to culture media (121). In the absence of Ubp2 activity, this modification to the ribosome is prolonged, continuing the resulting translation arrest (121).

Despite the broad range of pathways influenced and regulated by DUBs, the sensitivity of DUBs to oxidative stress has been established only within the past decade. As a result, there is much that remains to be explored to establish the full downstream impact DUB regulation by oxidative stress has on cellular physiology.

## DUBs as targets for therapeutic development

DUB involvement has been noted in a number of human diseases, in particular cancers and neurological disorders (37, 234). Many of these diseases become more prevalent as patients become older, due to accumulation of ROS from damaged mitochondria (235). In this scenario, prolonged oxidation of redox-sensitive DUBs may affect several physiological pathways, fostering the development and progression of human diseases. Oxidative stress and modulation of DUB activity have been specifically linked to certain neurological disorders, including Alzheimer’s disease, Parkinson’s disease, and epilepsy (36, 234, 236, 237). The involvement of diminished DUB activity in these neurodegenerative disorders may be due in part to the importance of ubiquitin signaling in regulating synaptic function (36). Many of the DUBs known to be involved in cancers are also redox-sensitive, such as USP7, which has been implicated in liver cancer and leukemia, and A20, which is involved in metastatic cancers (238, 239, 240, 241). Although much remains to be determined as to the specific roles DUBs play in these diseases and the impact of oxidative stress on them, it is undeniable that their malfunction leads to deregulation of critical pathways within neurons, making them important targets for the development of therapeutics to treat these conditions.

To date, more than 50 DUB inhibitors have been discovered, several of which are in preclinical development (49). Generation of small-molecule inhibitors specific for individual DUBs or DUBs that serve similar functions is a priority for the field. For this reason, it is critically important to study the pathways and interactions DUBs influence. One key challenge for developing specific DUB inhibitors is the similarities in catalytic and substrate interactions domains. General inhibitors would block the activity of multiple DUBs simultaneously, potentially impacting a broad range of cellular pathways. For this reason, noncompetitive inhibitors of DUBs are more ideal, since these can cause structural changes that impact the active/inactive state, the affinity for substrate, or the specificity of the DUB.

Another challenge for targeting DUBs for therapeutics is the potential for off-target effects. Identifying the different substrates that will be influenced by regulation of the DUB helps to identify potential side effects and consequences of inhibitor treatment to better select target DUBs and the inhibitors that produce the desired effects. DUB–substrate interactions are often transient, thus highly specific DUB inhibitors coupled to mass spectrometry analyses have been used to identify the substrates affected by the DUB (242). Identification of targets can be achieved by using substrate traps or through quantification of cellular proteins, identifying those that are destabilized upon DUB inhibition (243, 244). This methodology could be broadly applied to identify DUB substrates under a variety of *in vivo* conditions in order to expand the known roles of DUBs and therefore ubiquitin signaling in cellular physiology.

In 2015, the first therapeutic DUB inhibitor entered clinical trials for treatment of multiple myeloma through dual inhibition of USP14 and UCHL5. Dose toxicity remains a challenge for this drug (VLX1570); however, its identification and characterization represented a massive step forward in the DUB therapeutic field (245, 246). Indeed, this push for DUB therapeutics has also greatly furthered the field of DUB research in general, leading to the identification and elaboration of specific substrates and mechanisms. As discussed above, dysregulation of USP30 can lead to retention and accumulation of dysfunctional mitochondria, a hallmark of Parkinson’s disease (186). Recently, a USP30 inhibitor, ST-539, was demonstrated to be utilized for tissue-specific induction of mitophagy without impacting the function of the tissue (247). Studies such as this one are necessary to continue the drive for successful development of therapeutic DUB inhibitors.

## Conclusion

Much remains to be learned about how DUB activity is regulated, particularly regarding the impact of oxidative stress, as some DUBs are more susceptible to oxidative stress and reversal of cysteine oxidation through DTT treatment (35). Further research may demonstrate that these differences result from particular structural features that make DUBs more or less susceptible to oxidation and/or reduction. The type of ROS and the location of their subcellular production could also differentially affect DUBs, which would increase the regulatory capacity of this mechanism. Protein interaction networks may play a role, by sequestering or recruiting DUBs from or to stress response processes. Furthermore, the timing, along with the tissue-specific expression of a DUB could impact its activity and exposure to stress.

Another critical point is understanding the physiological reversibility of DUB redox regulation. Although the artificial reducing agent DTT has been largely used to demonstrate the reversibility of DUB activity following oxidation, many questions remain open regarding the enzymatic system responsible for reducing DUB cysteine *in vivo*. This additional layer of regulation will help us fully comprehend their modes of regulation and their cellular impact during stress progression. Further complexity to the impact of ROS on the system is added when one considers that the E2 and E3 ubiquitin enzymes also utilize catalytic cysteines and are themselves variably sensitive to oxidation (77), creating a complex system to regulate levels of protein ubiquitination and function under stress.

Advances in proteomics, transcriptomics, and structural biology continue to be critical to elucidating the mechanisms behind redox regulation of DUBs and provide a better resolution of DUB catalytic processes and their interaction and expression profiles before, during, and after stress.

Understanding how individual DUBs are reversibly oxidized will enhance our knowledge of ubiquitin signaling regulation. With such knowledge, targeted mutations, such as the Ser510Gly mutation in USP19CD, could enable experimentation where the DUB functions normally until briefly exposed to low levels of oxidative stress, whereupon it is no longer able to regain function (35). Such studies could expose the acute effects of DUB loss.

Knowing what makes certain DUBs more resistant to oxidative stress than others could aid in our establishment of therapeutic targets and mechanisms to combat the effects of oxidative stress in human patients. For example, oxidative stress has been implicated in a number of neurodegenerative diseases, as have certain DUBs (234, 248). Knowing more about how DUBs react to oxidative stress could elucidate which DUBs involved in these diseases are primarily impacted by oxidative stress prior to disease onset. It may then aid in the discovery of new methods of treatment that enhance the resistance of the DUBs to stress.

DUBs utilize mechanisms that are often complex interconnected, and they often influence multiple cellular pathways, adding new challenges to the production of selective therapies. However, continued research of inhibitors, combined with advancements in target and selectivity screening and phenotypic analysis are providing steady progress toward successful development of a therapeutic DUB inhibitor. Development of inhibitors can be hastened by discovering more about what gives individual DUBs their unique properties, such as the variability of redox sensitivity.

Knowing the characteristics that make DUBs more or less susceptible to redox reactions could help identify which DUBs will be better candidates as therapeutic targets. DUB inhibitors may be able to target hyperactive DUBs, but other diseases can often result from a reduction of DUB activity, through mutations, hyperactive regulators, or oxidative stress. In these cases, therapies are needed to enhance DUB activity or their downstream pathways. For example, a previous review from Pfoh *et al.* (2015) (190) proposed the development of a drug that would block interaction between MDM2 and USP7 in patients with cancer caused by hyperactivation of MDM2, thereby targeting MDM2 for degradation and stabilizing p53.

By studying the DUBs and the pathways that are affected by their redox regulation, the upstream and downstream effectors that are deregulated due to DUB inactivity can be identified, and those can also become targets of therapeutic development. Due to the interconnectedness of DUB regulatory mechanisms, another DUB that stabilizes a hyperactive regulator may be identified as a potential target. Alternatively, allosteric drugs could be made to modify a DUB that functions similarly to broaden its activity or specificity to compensate for that of the affected DUB. Research could also uncover new ways to support the DUBs and help them to regain their activity by protecting them from stress or regulators through activation or inhibition of proteins in pathways related to DUB activation or inhibition. DUBs are central regulators to numerous cellular pathways, and by understanding how they function, what substrates they target, and how their activity is regulated, we can enhance our ability to treat disorders caused by their dysfunction.

## Conflict of interest

The authors declare that they have no conflicts of interest with the contents of this article.
